# Autophagy: Dual Response in the Development of Hepatocellular Carcinoma

**DOI:** 10.3390/cells8020091

**Published:** 2019-01-28

**Authors:** Hamza O. Yazdani, Hai Huang, Allan Tsung

**Affiliations:** 1 Department of Surgery, University of Pittsburgh, Pittsburg, PA 15213-2582, USA; obaidh3@upmc.edu; 2 Division of Surgical Oncology, Department of Surgery, The Ohio State University Wexner Medical Center, N924 Doan Hall, 410 West 10th Ave., Columbus, OH 43210, USA; huang.3900@osu.edu

**Keywords:** hepatocellular carcinoma, inflammation, mitophagy, oxidative stress, HCC therapy

## Abstract

Autophagy is an evolutionary conserved intracellular mechanism which helps eukaryotic cells in maintaining their metabolic state to afford high-efficiency energy requirements. In the physiology of a normal liver and the pathogenesis of liver diseases, autophagy plays a crucial role. Autophagy has been found to be both upregulated and downregulated in different cancers providing the evidence that autophagy plays a dual role in suppressing and promoting cell survival. Hepatocellular carcinoma (HCC) is the most common primary liver cancer and the major leading cause of cancer mortality worldwide. In light of its high complexity and poor prognosis, it is essential to improve our understanding of autophagy’s role in HCC. In this review, we summarize the dual mechanism of autophagy in the development of HCC and elucidate the currently used therapeutic strategies for anti-HCC therapy.

## 1. Introduction

Autophagy (macroautophagy) is the fundamental cellular process in maintaining cell homeostasis by targeting damaged intracellular organelles and misfolded proteins to lysosomal degradation [1]. It is a conserved evolutionary process that takes part in all mammalian cells under basal conditions and generates building block molecules to support essential cellular processes [2]. Autophagy is a multistep process including membrane rearrangement in forming a double-membrane bond structure known as autophagosomes. The vesicle fusion of these autophagosomes with lytic compartments generates autolysosomes where lysosomal enzymatic degradation of contents is recycled and releases nucleotides, fatty acids, and amino acids to refuel the cells with energy to maintain necessary molecular synthesis [2].

The role of autophagy is complex and differs from organ to organ. An organ such as muscles and the liver requires autophagy to remove excessive protein aggregation, lipid accumulation, and damaged mitochondria to prevent excessive ROS generation leading to oxidative stress [3,4]. Defects in autophagy have shown to contributes in several pathogeneses of human diseases ranging from neurodegenerative and metabolic diseases to cancers [5]. The dysregulation of autophagy has been increasingly indicated to play a role in liver diseases such as alcoholic liver disease, non-alcoholic fatty liver disease (NAFLD), hepatosteatosis, hepatomegaly, and primary liver malignancies such as hepatocellular carcinoma (HCC) [6,7].

Hepatocellular carcinoma is a serious threat towards human health. It is the sixth most malignant cancer worldwide and the fifth most common malignancy in men [8]. Despite recent advances in treatments and surgical resection, the five-year survival rate remains unsatisfactory [9]. The most common identified risk factors for HCC development are the consequences of unresolved oxidative stress, persistent inflammation, viral infections, metabolic dysfunction, liver alcohol disease, and fatty liver disease. Autophagy may serve as a protective mechanism against the initial and persistent liver injury in these disease states but autophagy may also play a significant role in the development and growth of hepatic tumor cells in this inflammatory environment [10,11]. The link between autophagy and cancer has been long proposed. The underlying mechanisms regulating the autophagic response in HCC requires further understanding to develop effective treatment strategies. 

## 2. Role of Autophagy in Normal Liver Homeostasis

Autophagy is involved in diverse physiology and pathophysiology of the liver. The liver displays a complex metabolism with a variety of functions including protein and lipid synthesis and secretion of bile acid. Increased accumulation of ubiquitin proteins aggregation observed in the liver-specific Atg7 knockout mice suggested a basal function of autophagy in continuous turnover of the cytoplasmic proteins [12,13]. Disturbance in the basal autophagy of the liver can lead to the accumulation of elementary bodies, damaged mitochondria, deformed peroxisomes, and abnormal membrane structures resulting in liver injury. Autophagy can be general and nonselective, involving the degradation of a bulk cytoplasmic portion or organelle-specific degradation. The cell can undergo different forms of autophagy which include xenophagy (degradation of viruses), lipophagy (degradation of lipid droplets), ribophagy (degradation of ribosomes), pexophagy (degradation of peroxisomes), reticulophagy (degradation of ER), and mitophagy (degradation of mitochondria). Of these, mitophagy is one of the most well characterized since hepatocytes contain numerous mitochondria to provide the high energy demand for metabolism. Liver specific autophagy deficient mice provide evidence for swollen mitochondria and increased ROS formation [14]. Liver injury is also associated with mitochondrial membrane permeabilization which can activate a mitochondrial apoptotic pathway regulating BAX and Bad (BCL2 family) mediated cell death [15]. The different forms and role of autophagy in the healthy liver are best described in detail by Takashi U Eno et al. [16].

Hepatocytes are primarily dependent on autophagy degradation due to its intense metabolism and high energy demand to maintain proper functioning. The defect in any of the forms of autophagy can contribute to severe liver functional damage such as hepatitis, fibrosis, cirrhosis, and HCC development [17,18]. One of the most common causes of acute and chronic liver disease is the infection of the liver with hepatotropic viruses. Hepatitis B and C viruses are both linked to the autophagy. It has been shown that the X protein of the HBV (HBx) can induce autophagy due to its ability to bind to the PI3k autophagic molecule. On the other hand, the Hepatitis C virus (HCV) can adapt autophagy content to enhance its replication by inhibiting the maturation of autophagosome into autophagolysosomes. Both these viruses are shown to induce autophagy by transcriptional upregulation of Beclin1 [19].

Autophagy also plays a role in lipid metabolism and thus can influence the development of fatty liver disease, a rapidly increasing cause of chronic liver disease in the world. Hepatocytes are the main source for natural lipids stored in the form of triglycerides (TGs) and lipid droplets (LDs). Interestingly, the first degradation of lipid droplets through lipophagy was observed in mouse liver. It has been shown that ATg7 knockout mice showed significant increase levels of TGs and LDs in the hepatocytes leading to a decrease in the levels of free fatty acids (FFAs), necessary for ATP generation [20]. Another study illustrates the formation of hepatic steatosis in mice due to the consumption of a high-fat diet. This process occurs through autophagy activation in hepatic stellate cells and is due to the suppression of mTOR phosphorylation which increased expression of Sirt1 through the AMPK pathway [21].

## 3. Role of Autophagy in Hepatocellular Carcinoma

Hepatocellular carcinoma is one of the most common primary cancer and a major leading cause of death worldwide [22]. An estimation of 30,200 deaths is predicted this year [23]. The high proliferative capability of HCC cells has been linked to persistent inflammation and increased oxidative stress. Autophagy has been shown to play a dual role in cancer. Autophagy could be either tumor suppressive or tumor promoting. Here we review both the pro- and anti-tumor mechanism of autophagy that take place during the development and growth of HCC.

### 3.1. Tumor-Suppressive Role of Autophagy 

Inflammation is recognized as a hallmark of cancer in promoting tumor growth and is associated with the poor prognosis of many solid tumors [24]. It is well known that HCC progression is highly correlated with the persistent inflammatory stimulation [25]. Autophagy has been suggested to prevent cancer progression by suppressing inflammation [26,27,28,29] while inhibition of autophagy can lead to a sustained and elevated level of inflammation [30,31]. Inflammasomes are the major drivers for chronic inflammation in the liver [32,33]. Interestingly, the capability of cells to suppress inflammation through autophagy was first observed in autophagic inhibited mice. Lack of autophagic responses in ATG16L1 knockout mice undergoing septic shock revealed an elevated level of inflammasome associated IL-1β and IL-18 cytokine production compared to wild-type controls [28]. Further studies showed that depletion of the autophagic proteins, LC3B and Beclin1, enhanced Caspase-1 activation which was NALP3 inflammasomes dependent. Mice lacking LC3B protein were more susceptible to LPS induced mortality [34]. In addition, a recent study showed the effect of chemokine (C-X-C motif) ligand 17 (CXCL17) in suppressing autophagy [35]. In human HCC tissues, elevated expression of CXCL17 has been observed which can also promote cell proliferation and migration when treated invitro. Silencing of this induced autophagy which is due to the enhanced nuclear translocation of liver kinase b1 (LKB1) that phosphorylates and activates AMPK. This lead to decrease in tumor volume and proliferation.

Beclin1, an important autophagic protein, has been shown to be associated with HCC tumors. The decreased Beclin1 expression observed in human HCC tissues correlated with tumor recurrence and disease-free survival [36]. Studies have also shown that knocking out Beclin1 in mice is embryonically lethal whereas heterogeneous Beclin1 mice developed spontaneous HCC [37]. In addition, normal hepatocytes show higher expression and activity of autophagic-associated proteins compared with HCC cell lines providing evidence that autophagy plays a tumor suppressive role [14]. A recent study demonstrated the effect of adrenaline in promoting hepatocarcinogenesis [38]. Treatment of adrenaline in DEN-induced HCC mice showed a remarkable increase in liver injury along with increased tumor number and tumor size. Mechanistically, increase in adrenaline activated the adrenergic receptors ADRB2 which inhibited autophagy activation by disrupting the Beclin1/VPS34/ATg14 complex.

Since autophagy plays a vital role in suppressing cancer inflammation, inhibition of autophagy has also been shown to result in an excessive accumulation of p62 protein [39]. P62 is an autophagic substrate which is used in measuring autophagic activity [40,41]. Transgenic P62 knockout animals displayed irregular cell mitotic activity with an increased expression of insulin-like growth factor 2 (IGF2) in their HCC tumors [42]. It has been reported that p62 is necessary for the HCC initiation by maintaining metabolic homeostasis through the mTOR pathway [43] and ablation of p62 inhibits growth and proliferation [14]. In a study, 90 HCC-resected tumors were analyzed for the accumulation of intracellular hyaline bodies (IHB) which are cytoplasmic inclusions consisting of p62. Patients with increased IHB were shown to be associated with significantly shorter overall survival [44]. Moreover. P62 is also known to interact with tumor necrosis factor receptor-associated factor 6 (TRAF6) that induces nuclear factor (NF-kB) activation [45]. Zhang et al. [46] have shown that DEAD-box protein 5 (DDX5), a tumor suppressor protein in the liver [47], binds to p62 and interferes with P62/TRAF6 interaction. This results in autophagy induction. However, the expression of DDX5 in the human HCC tumor tissues is relatively lower when compared to its non-tumor counterpart. Autophagy has also been shown to contribute to the anti-proliferative activity of interferon gamma (IFN-gamma), a pleiotropic cytokine that facilitates anti-viral and anti-proliferative effects in cancer cells. In HCC cell lines, stimulated autophagosome formation inhibited cell growth. Silencing of autophagy in these cells abolished the inhibitory effect, suggesting an essential anti-tumorigenic activity [48].

Similarly, multiple miRNAs which are found to target autophagic comprised genes for autophagy modulation [49,50,51] may also play a part in HCC growth and inhibition. miR-7 is a well acknowledged tumor suppressive microRNA in cancers. In HCC tumors, levels of miR-7 are significantly downregulated. It has been shown that upregulation of miR-7 in HCC cell lines increases autophagic activity by targeting the mTOR pathway, leading to a decrease in cancer cell proliferation [52]. miR-85 is an essential component during liver development and has also been linked as a tumor suppressor in HCC. In human HCC cell line HepG2, transfection of miR-85 upregulated autophagic activity which resulted in cell cycle arrest [53]. Our group has also previously shown that miR-375 is downregulated in HCC cell lines and tissues [54]. We found that miR-375 inhibits autophagy by suppressing the conversion of LC3I to LC3II by downregulating ATG7. Inoculating miR-375 overexpressed cells in nude mice showed significantly decreased tumor growth [51].

### 3.2. Tumor-Promoting Role of Autophagy

Hypoxia-induced oxidative stress is one of the most prominent features in all solid tumors [55,56] due to the inadequate blood supply that tumors experience as they grow. Under oxygen deprivation, cells respond by regulating their metabolic and bioenergetics demands to overcome the hypoxic resistance [57,58,59]. Hypoxia-induced autophagy in hepatocytes and HCC tumor cells relies upon the stabilization of hypoxia-inducible factor (HIF1α). HIF is the important regulator that maintains oxygen homeostasis. It has been shown that HIF1-α upregulates BNIP3 and BNIP3L proteins which binds to the BCL-2 protein. This process inhibits the disruptive interaction between BCL-2 and Beclin1 [60] to induce autophagy for cellular survival in hypoxic conditions. In addition, hypoxia is also shown to upregulate early growth response gene-1 (Egr-1) expression. Egr-1 is a zinc finger nuclear protein and functions as a transcriptional regulator [61]. Upregulation of hypoxia induced Egr-1 has shown to induce autophagy in HCC cell lines promoting migration. Inhibiting Egr-1 function by Ad-DN-Egr-1 revealed the attenuated autophagosome formation due to inhibited binding of Egr-1 at LC3 promoter region (−233 to −214) [62].

The extensive reactive oxygen species generation during the developing tumor is the primary outcome of hypoxic stress. Increased ROS levels are shown to oxidize cellular components such as DNA, lipids, and proteins [63,64]. ROS can be generated by the NADPH oxidase complexes in the cell membrane, endoplasmic reticulum, peroxisomes, and mitochondria [65,66]. Tumors adapt different mechanisms to eliminates intracellular ROS, one of which is by the upregulation of anti-oxidant proteins NRF2 [67]. NRF2 is a cytoplasmic protein which translocates to the nucleus for the transcription of redox-balancing proteins and β-oxidant enzymes and is upregulated by autophagy. The autophagic protein p62 has been shown to interact with NRF2 [68]. Phosphorylated p62 binds with Keap1 (NRF2 inhibitory protein) allowing NRF2 release and cytosolic stabilization [69,70]. The clinical implication of NRF2 was elucidated in 107 HCC patients where patients that expressed higher levels of pNRF2 were significantly associated with worst disease-free survival and poor overall survival [71]. Thus, p62 induces the antioxidant activation pathway to prevent extreme organelle injury leading to tumor cell death.

Another important mechanism to control excess ROS production is by the removal of damaged organelles from the cell. Injured and non-functional mitochondria are the major source of cellular ROS production and the induction of mitophagy helps clear these damaged mitochondria to maintain cell function and bioenergetics [72,73]. Mitochondrial ROS is eliminated regularly by the superoxide dismutase within the cytosol [74,75] which is also activated by the NRF2 protein [76]. Depolarized mitochondria cleared via mitophagy are regulated by PINK1 and Parkin1 [77]. It is reported that mitophagy can degrade tumor suppressor p53 once localized to mitochondria and inhibit its subcellular localization. Mitophagy impairment results in p53 accumulation and its phosphorylation by PINK1 prevent hepatic cancer cell stemness [78]. However, excessive induction of mitophagy can also inhibit HCC migration. When yes-activated protein (Yap), a highly upregulated protein in HCC, is depleted, mitophagy is overactivated leading to cellular energy deprivation [79]. In contrast, the excessive activation of autophagy may also lead to autophagic cell death [80,81]. This process requires an additional cell death signaling, including the phosphorylation of c-Jun N-terminal kinase (JNK) [82]. Interestingly, the pro-apoptotic (Bax/Bak) deficient cells showed an autophagic cell death when exposed to apoptotic stimuli. This was driven through the increase in the level of JNK phosphorylation. However, treatment with the JNK inhibitor further revealed that a simultaneous activation of autophagy and JNK is required for the autophagic cell death. Numerous other cell death and tumor suppressor-related proteins are shown to trigger autophagic cell death in the literature [83,84,85].

Autophagy can also regulate inflammatory immune response by the release and degradation of damage associated molecular patterns (DAMPS) including high mobility group box 1 (HMGB1), histones, ATP, mitochondrial (mt)DNA, and mitochondrial transcription factor A (TFAM). HMGB1 is a well-characterized DAMP which can be released from necrotic or apoptotic hepatocytes [86,87]. Interestingly, autophagy-mediated intracellular mobilization of HMGB1 enables tumor growth by inducing cell survival and apoptosis [88,89]. We have previously shown that under hypoxic stress, HMGB1 translocates from nucleus to cytoplasm in HCC cells. Intracellular translocation of HMGB1 facilitates its interaction with mtDNA in the cytosol where together they activate TLR9 signaling pathways to enhance tumor growth [90].

### 3.3. Autophagy in HCC Metastasis

Autophagy, due to its tumor promoting role, also plays a part in tumor metastasis. Increasing evidence suggests the upregulation of autophagy during the process of tumor metastasis [91,92]. As the primary tumor grows and encounter harsh microenvironment, part of it escapes and intravasates within the circulatory and lymphatic system, localizing at distal organs [93]. Cell detachment from the extracellular matrix (ECM) has demonstrated to trigger autophagy which then protects it from anoikis, a type of cell death induced in response to ECM detachment [94]. In addition, Autophagy can also induce changes in the cell adhesion signaling that facilitates invasion and migration [95].

Autophagy in hepatocellular carcinoma has shown to facilitate metastasis by upregulating the expression of epithelial–mesenchymal transition (EMT). Induction of autophagy in the starved cells inhibited the expression of epithelial markers and induced mesenchymal expression along with cell invasion marker matrix metalloproteinase-9 (MMP9) [96]. These changes were regulated by the activation of TGF-beta and phosphorylation of Smad3 signaling pathway. In another study, inhibition of autophagy via silencing Beclin1 and ATG5 in HCC lung metastasis model markedly decreased distal metastasis to the lungs [97]. This effect is observed due to the impaired anoikis resistance that can suppress the colonization of HCC cells. However, silencing of autophagy mediators showed no effect in the expression of cell migration and invasion regulators.

Autophagy-mediated cancer cell metastasis can be stimulated by numerous stress factors persisting within the tumor microenvironment. One of which is the fluid shear stress (FSS). Wang et al. [98] showed that HCC cells, when exposed to 1.4 dyn/cm^2^ of FSS, induce autophagy in a time-dependent manner. Furthermore, inhibition of autophagy attenuated cells to migrate and downregulate the expression of PI3K/FAK/Rho GTPase pathway. This suggests that the activation of autophagy via FSS is a PI3K/FAK/Rho GTPase-dependent pathway.

## 4. Autophagy in HCC Therapy

As described above, autophagy can be either be pro- or anti-tumorigenic for HCC. Therefore, modulation of autophagy-based HCC therapy is very complex and will vary depending on the setting. Autophagy can suppress liver inflammation and thus decrease the carcinogenic environment in the liver but it can also promote and maintain tumor cell homeostasis by inducing mitophagy in growing HCC tumors. In addition, both the induction and inhibition of autophagy has been investigated to induce tumor cell death [99,100]. Currently, there are a variety of therapeutic agents that are being used either individually or in combination with other agents for anti-HCC therapy. The current agents used to selectively target autophagy to induce HCC cell death are described in Table 1.

### 4.1. Autophagy Inducers

The autophagy inducer sorafenib is the first-line drug used for the treatment of advanced HCC [101]. Currently, sorafenib is the only drug that has been shown to improve HCC patient survival [102]. In a randomized controlled trial, patients with advanced HCC treated with sorafenib had an improved rate of overall survival compared to patients given placebo. Sorafenib has been shown to promote cell death through the upregulation of autophagy via myeloid cell leukemia-1 (Mcl-1) signaling pathway [103]. In addition, sorafenib can also inhibit tumor growth by targeting the RAF/MEK/ERK pathway to induce cell cycle arrest [104]. Besides Mcl-1 and ERK pathways, sorafenib can also inhibit the activation of PI3K/AKT/mTOR pathways which initiates a signaling cascade for autophagy induction [105,106]. In a recent study, sorafenib is also shown to regulate cell endoplasmic reticulum (ER) stress, JNK, Akt, and AMPK pathway leading to elevated autophagy which later shifts towards apoptosis [107]. At an early time point (3–12 h) sorafenib increases ER stress which is shown to induce the autophagic survival process in HCC cell line by regulating JNK/AMPK signaling pathway. At a later stage (24 h), a significant increase in the ER stress and PERK-CHOP dependent rise of Bim shifted autophagy to apoptosis cell death.

Besides its tumor suppressing function, long term sorafenib treatment has also shown to trigger chemo-resistance in HCC cells [108]. Thus, the combination of sorafenib with SAHA, another autophagy-inducer, has been used to improve responses against HCC compared to treating with sorafenib alone [109]. Sustained treatment of sorafenib has also shown to increase tumor hypoxic environment that leads to decrease treatment efficiency [110]. Combination of melatonin with sorafenib has shown to enhance sorafenib’s cytotoxicity against human HCC cells by decreasing hypoxic resistance [111]. Co-administration diminished the expression of BNIP3 and NIX, hypoxia induced mitophagy mediators. Due to the common occurrence of tumor resistance, the combinations of different drugs with sorafenib are currently being studied to increase chemo-sensitivity and decrease tumor growth. A detailed combination of reagents with sorafenib are extensively reviewed elsewhere [112].

### 4.2. Autophagy Inhibitors

Since autophagy can be utilized by the growing cancer cells for its survival and add resistance towards chemotherapeutic reagents, inhibiting autophagy can also be a promising strategy against cancer therapy. At present chloroquine (CQ), an anti-malarial drug, can be used for the inhibition of autophagy through the suppression of lysosomes. Treatment with CQ neutralizes the pH levels of lysosomes required in the final stages of autophagy for degradation [113]. CQ is being effectively used in patients with the combination of drugs capable of inducing cell apoptosis such as oxaliplatin [114]. It is reported that co-treatment of proliferative HCC cell lines with CQ and sorafenib can lead to a marked suppression of its growth [105]. In a liver xenograft tumor model, nude mice treated with CQ and sorafenib combined achieved a higher level of tumor regression compared to treatment with sorafenib alone. In addition, 3-methyladenine (3-MA) which inhibits the interaction of autophagosomes and lysosomes can enhance anti-HCC therapy when combined with cisplatin, doxorubicin, and sorafenib [115]. Similarly, suppression of autophagy by administration of 3-MA and inactive ATg4B suppresses the proliferation of Huh7 cells [116]. Hence, inhibition of autophagy can promote the death of HCC cells but these treatments with their known side effects highlight a major risk in triggering the neoplasticism within the normal hepatocytes.

## 5. Concluding Remarks

**Figure 1:**
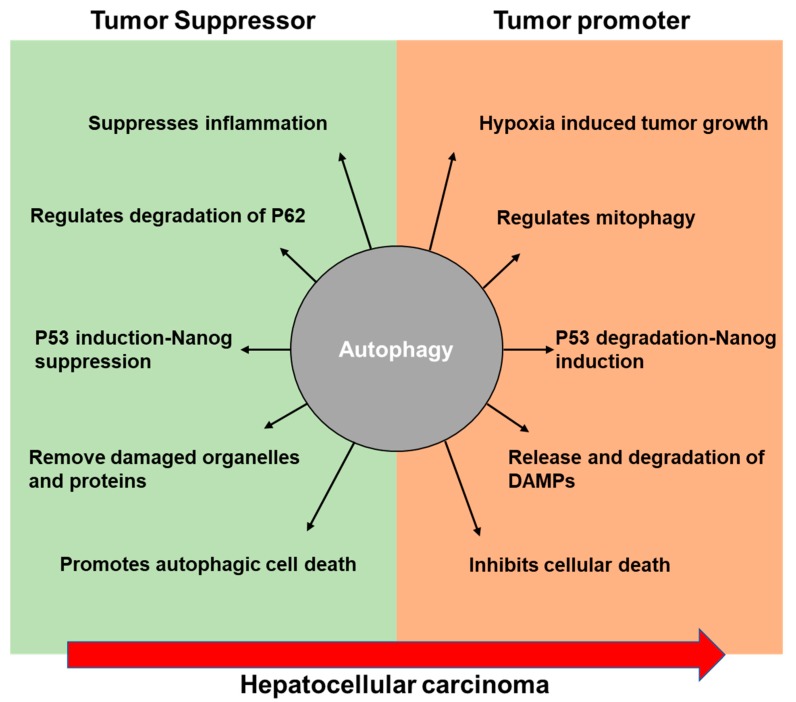
Schematic diagram illustrating the proposed role of autophagy during the development of hepatocellular carcinoma.

Autophagy is the vital response for hepatocytes undergoing stress in the maintenance of cellular homeostasis and quality control. As we outlined in this review, evidence supports a dual role of autophagy in the progression of HCC (Figure 1). Targeting autophagy can be used to treat liver cancer. However, caution should be noted as autophagy can inhibit the development of HCC but also act as a tumor promoter resulting resistance to HCC tumor therapy [137]. Multiple direct and indirect interactions including complex mechanistic overlap between apoptotic and autophagic cell death have been studied [138,139]. Many studies indicate that the apoptotic events alter autophagy. However, the molecular role of autophagy controlling apoptosis requires further investigation. In the developing tumor microenvironment, hypoxia is a common stimulus in many cancers and hepatotropic viruses can induce a hypoxic environment initiating hypoxic oxidative and inflammatory responses [140]. Thus improving tumor oxygenation or suppressing hypoxia-induced autophagy signaling could be a potential future therapeutic pathway to target. We currently have a minimal understanding regarding the stress signaling that takes part in the complex coordination of autophagy to respond during different intracellular and extracellular stimuli in HCC. Additional studies are necessary to further elucidate the mechanisms underlying autophagy’s role in HCC.

## Figures and Tables

**Table 1:** Therapeutic reagents modulating autophagy in anti-HCC treatment.

Reagents	Autophagy Target	Cells Treated	Result	Ref.
Sirolimus (Rapamycin)	mTOR	HepG2	Upregulation of autophagy-cell death	[117,118]
Temsirolimus (CCI-779)	mTOR	HepG2, Huh7	Upregulation of autophagy-cell death	[119]
Everolimus (RAD001)	mTOR	Hep3B, HepG2, Huh7	Upregulation of autophagy-cell death	[120]
Pemetrexed	mTORC1	HepG2	Upregulation of autophagy-cell death	[121]
SC-59	mTORC1	PLC5, Sk-Hep1, HepG2 and Hep3B	Upregulation of autophagy-cell death	[122,123]
BEZ235	PI3K/mTOR	Hep3B, PLC/PRF/5	Upregulation of autophagy-cell death	[124]
MK-2206	AKT	SNU449, SNU378, SNU475	Upregulation of autophagy-cell death	[125]
SB203580	MAPK	HepG2, Hep3B, PLC/PRF/5, Huh-7	Upregulation of autophagy-cell death	[126]
Regorafenib	Tyrosine-kinase inhibitor	HepG2 and Hep3B	Upregulation of autophagy-cell death	[127]
Sorafenib	Tyrosine-kinase inhibitor	Hep3B, HepG2, Huh7	Upregulation of autophagy-cell death	[105,112]
Nilotinib	Tyrosine kinase inhibitor	PLC5, Huh-7, Hep3B	Upregulation of autophagy-cell death	[128]
ABT-737	JNK pathway	Huh7, HepG2	Upregulation of autophagy-cell death	[129]
OSU-03012	PDK1/AKT	Huh7, Hep3B, and HepG2	Upregulation of autophagy-cell death	[130]
5-FU	Induce ER stress	HepG2, SMMC-7721, Hep3B, BEL-7402	Upregulation of autophagy-cell survival	[114]
Bortezomib	Proteasome inhibitor	Huh7, HepG2, HuH7, human hepatocytes, rat hepatocytes	Upregulation of autophagy-cell death	[131]
Chloroquine	Lysosome	HepG2, Huh7, HA22T/VGH, Mahlavu	Downregulation of autophagy-cell death	[132,133]
3-MA	PI3K III	H22, HepG2, PLC/PRF/5, SMMC7721	Downregulation of autophagy-cell death and cell survival	[134,135]
Bafilomycin A1	Lysosome	BEL7402, HepG2, Huh7, SMMC-7721	Downregulation of autophagy-cell death	[136]
